# Plasma levels of miR-21b and miR-146a can discriminate rheumatoid arthritis diagnosis and severity

**DOI:** 10.37796/2211-8039.1637

**Published:** 2025-03-01

**Authors:** Rizk S. Sarhan, Amr M. El-Hammady, Yasmin M. Marei, Sania K. Elwia, Doaa M. Ismail, Emtethal A.S. Ahmed

**Affiliations:** a Department of Internal Medicine, Faculty of Medicine, Benha University, Benha, Egypt; b Department of Medical Biochemistry & Molecular Biology, Faculty of Medicine, Benha University, Benha, Egypt; c Department of Physical Medicine, Rheumatology & Rehabilitation, Faculty of Medicine, Tanta University, Tanta, Egypt; d Department of Physical Medicine, Rheumatology & Rehabilitation, Faculty of Medicine, Benha University, Benha, Egypt

**Keywords:** Rheumatoid arthritis, Osteoarthritis, microRNA-146a, microRNA-21b, Early arthritis

## Abstract

**Objectives:**

This study tried to examine the ability of the estimated plasma gene-expression levels (PGEL) of microRNA (miR)-146a and miR-21b to distinguish patients with early rheumatoid arthritis (RA) out of arthritis patients who did not fulfill the diagnostic spectrum of either RA or osteoarthritis (OA); the diagnostic Gray-Zone (GZ).

**Patients & methods:**

Enrolled patients underwent full diagnostic workup and were categorized as highseropositive and fulfilled the diagnostic spectrum for RA (RA-group), seronegative and fulfilling the diagnostic spectrum of OA (OA-group) and low-seropositive or seronegative patients who did not fulfill diagnostic criteria of RA or OA (GZ-group). Blood samples were obtained for quantification of PGEL of miR-146a and miR-21-b using the quantitative Reverse-transcriptase polymerase chain reaction and results were related to patients’ seropositivity and clinical data.

**Results:**

The mean fold change of PGEL of miR-146a and miR-21b was significantly higher in patients than in control samples, in samples of high-seropositive patients than in other samples, and in samples of low-seropositive than in seronegative patients. Both markers showed a positive significant correlation with Disease Activity Score-28 for RA-activity and seropositivity. Using the ROC curve analysis, the PGEL of both microRNAs could identify high-seropositive among the studied arthritis patients, but Regression Analysis defined high PGEL of miR-146a as the most significant predictor to identify RA patients and predict their disease activity. Statistical analyses defined miR-146a as the significant parameter that could differentiate between early RA and OA patients among GZ patients.

**Conclusion:**

Early arthritis that does not fulfill the diagnostic spectrum of a certain type of arthritis is not uncommon and challenges therapeutic decision-making. The estimated PGEL of MicroRNA-146a might enlighten this gray diagnostic zone and allow differentiation between patients with early RA and early OA, and help to stratify RA patients according to disease activity and severity.

## 1. Introduction

Chronic knee pain (CKP) is a common musculoskeletal condition, which usually causes increasing levels of functional deficits and mobility issues with decreased quality of life (QOL) and substantial financial burden [1].

Rheumatoid arthritis (RA) is an autoimmune, chronic, and progressive inflammatory disease affecting approximately 1% of the global population with female predilection [2]. RA is characterized by chronic inflammation and oxidative stress that mainly targets the synovial membrane causing articular lubrication deficiency with excessive release of proinflammatory cytokines leading to disease progression with subsequent joint stiffness, deformity, and dysfunction [3]. A major proportion, about 62%, of clinically suspected RA patients were suffering from knee pain [4].

Osteoarthritis (OA) is a chronic joint bone disease, characterized by inflammatory destruction and hyperplasia of bone and mainly presents with pain and joint-mobility difficulties [5]. Globally, about 7.6% of the global population had OA with a 10-y increase of 132.2% in total cases and a 74.9% increase in knee OA (KOA) cases [6].

The increasing prevalence of CKP resulted in a wide diagnostic gray zone (GZ), where a large number of patients did not fulfill the diagnostic criteria for major types of arthritis [7]. Moreover, the documented variability of the diagnostic yield of the settled guidelines for arthritis [8] and the contradictory therapeutic strategies for OA and RA [9,10] necessitate finding early effective diagnostic biomarkers to unveil the diagnosis of patients of this gray zone.

MicroRNAs (miR) are noncoding small RNAs responsible for the regulation of multiple cellular processes, especially cell development, differentiation and proliferation [11]. However, aberrant expression of several microRNAs was found to play a pivotal role in various disorders and can serve as disease diagnostic biomarkers and/or therapeutic agents [12].

## 2. Hypothesis

This study suggested that the estimation of plasma gene-expression levels (PGEL) of micro-RNAs might distinguish patients with CKP who did not fulfill the diagnostic criteria for either RA or OA types of arthritis (GZ patients) and stratify RA patients according to disease severity.

## 3. Objectives

The study targets to define the diagnostic performance of the estimated plasma gene-expression levels (PGEL) of miR-146a and miR-21b for differentiation of CKP patients of the diagnostic GZ and to stratify RA-patients according to disease activity score-28 (DAS-28).

## 4. Settings

Departments of Internal Medicine, Rheumatology and Rehabilitation, Medical Biochemistry, Faculty of Medicine, Benha and Tanta Universities.

## 5. Design

Prospective comparative non-randomized multi-center study.

## 6. Ethical consideration

The departmental approval of the study protocol was obtained before case collection. The study protocol was fully discussed with each patient, and those accepted to give blood samples signed written consent. After complete sample collections, the final approval by the Local Ethical Committee at Benha Faculty of Medicine was obtained and registered by number: RC: 1-4-22.

## 7. Blindness

Internal medicine physicians evaluated patients clinically for the presence of the diagnostic criteria and severity of both RA and OA and the inclusion and exclusion criteria. Pain physicians were responsible for local clinical examination and evaluation of pain and disability scores. Medical biochemists underwent estimation of plasma expression levels of the studied microRNAs and were blinded about the indication of the estimation and whether the sample was a study or a control sample. Each physician was blinded about the results of other evaluations till the end of case collection. Thereafter, the PGEL of microRNAs were interpreted against the clinical and other lab data.

## 8. Patients

Patients who attended the outpatient clinics of the Internal Medicine and Rheumatology Departments with CKP from Jan 2020 to Feb 2022 were evaluated for age, gender, body mass index (BMI), presence of other general co-morbidities especially autoimmune or degenerative diseases, duration of symptoms, and type of received medications. Then; full general examination and clinical evaluation of all body joints was performed and radiologic diagnostic workup was performed. Controls were collected from those who attended the blood bank after passing clinical examination and investigations required for blood donation and were free of any manifestations of arthritis and exclusion criteria.

## 9. Evaluation tools (Appendix I [https://www.biomedicinej.com/cgi/editor.cgi?article=1637&window=additional_files&context=biomedicine])

1. RA was diagnosed and scored according to the 2010 RA classification criteria with a score of ≥6 was used as the cutoff point for RA diagnosis [13].
2. Disease activity score-28 (DAS28), which assesses four items; the number of swollen and tender joints, estimated ESR level and the global health assessment and the scores’ sum determined RA activity at cutoff point DAS28 ≥ 2.6 [14].
3. Clinical diagnosis of OA was performed according to the U.K. NICE 2014 Guidelines for Osteoarthritis of the Knee [15].
4. Pain severity was assessed using the numeric rating scale (NRS) with higher scores indicating severe pain [16].
5. Disability secondary to arthritis-induced pain or limited mobility was determined using the Oswestry Disability Index (ODI) with a higher total score indicating increased disability [17].
6. The impact of arthritis on patients’ QOL was evaluated by the self-administered Western Ontario and McMaster Universities Arthritis Index (WOMAC) which evaluates pain, stiffness, and physical function and higher scores indicate the worst impact of arthritis on patients’ QOL [18].

## 10. Exclusion criteria

Patients with arthritis other than RA or OA, and autoimmune disorders other than RA, maintained on immunosuppressive or disease-modulating drugs were excluded.

## 11. Inclusion criteria

Patients with manifestations suggestive of RA or OA and were free of exclusion criteria were enrolled in the study.

## 12. Sample size

The sample size was calculated by the G*Power (Version 3.1.9.2) [19] according to the previously published comparative work evaluating the PGEL of miR146a and miR-21b in RA cases versus control subjects [20,21]. The null hypothesis was considered as the absence of difference between groups regarding the PGEL of miR-146a and miR-21b. Considering the effect size of 0.20, using the F test model, the required sample size must be 52 subjects per group using α error of 5% and a power of 80% to ensure the uncertainty of the null hypothesis.

## 13. Lab investigations

Blood samples were aseptically collected and divided into two parts: one part was collected in EDTA tubes and preserved at −80 °C for the relative quantification of the PGEL of miR-146a and miR-21b. The second part was used for estimation of erythrocyte sedimentation rate (ESR, mm/h), serum levels of C-reactive protein (CRP in mg/L), and anticyclic citrullinated peptide (ACCP), and detection of seropositivity for Rheumatoid factor (RF).

## 14. Methodology

As described by **Yang et al**. [21] the following steps were conducted:

- Isolation of total RNA including microRNA was performed using the miRNeasy Mini Kit (QIAGEN, Germany). Synthesis of complementary DNA was performed using miScript II RT Kit (QIAGEN, Germany; IDs for hsa-miR-146a-5p and hsa-miR-21b, MIMAT0000449 & MIMAT0004608). The recommended RNA starting amounts and buffers for reverse transcription reactions for quantization of miRNAs were, by using miScript precursor assays, 5x miScriptHiFlex buffer and the recommended RNA input depends on the abundance and number of target miRNAs to be quantified; up to a maximum of 1 μg (miRNeasy Mini Kit, 2-miScript II RT Kit: QIAGEN, Catalog no. 217004, 218161 respectively). The mixture was incubated at 37 °C for 60 min and at 95 °C for 5-min to inactivate the miScript Reverse Transcriptase. Then, the mixture was placed on ice and diluted with 40 μl RNase-free water to the 10-μl reverse transcription reaction, mixed gently then briefly centrifuged and continued with quantitative real-time PCR for detection of microRNAs expression levels using QuantiTect SYBR Green PCR Kit. The PCR reaction mix was prepared in a total volume of 25 μl/tube (12.5 μl of 2x QuantiTect SYBR Green PCR Master Mix, 2.5 μl of 10x miScript Primer Assay, 2.5 ml of 10x miScript Universal Primer, Template cDNA up to 250 ng and RNase-free water).
- The real-time cycler was programmed using ABI 7900HT Fast Real-Time PCR System, (Applied Biosystem, Singapore) as initial activation for 15-min at 95 °C, denaturation at 94 °C for 15-sec, annealing at 55 °C for 30-sec and extension at 70 °C for 30-sec, for 40 cycles.
- MicroRNA expression levels in each sample were determined after correction with the GADPH expression level. Controls were chosen as the reference samples, and fold changes in the PGEL of microRNAs were determined by the 2^−ΔΔCT^ (cycle threshold) method and expressed as fold change (FC) using Step One software (Applied Biosystems, USA).

## 15. The sequences of the used primers

[Table t8-bmed-15-01-030]

**Table t8-bmed-15-01-030:** 

Items	Sequences
miR-146a-F	5′-CAGCTGCATTGGATTTACCA-3′
miR-146a-R	5′-GCCTGAGACTCTGCCTTCTG-3′
miR-21-F	5′-TGAGACTGATGTTGACTGTTGAA-3′
miR-21-R	5′-TGTCAGACAGCCCATCGAC-3′
GAPDH-F	‘5′-CCACCCATGGCAAATTCCATGGCA-3′
GADDH-R	5′-TCTAGACGGCAGGTCAGGTCCAC-3′

## 16. Study outcomes

1. The diagnostic performance of the PGEL of microRNAs to identify RA-patients among CKP patients who did not fulfill the clinical diagnostic criteria for either RA or OA
2. The relation between the PGEL of microRNAs and patients’ clinical data and DAS28 for RA patients.

## 17. Results

**Fig. 1:**
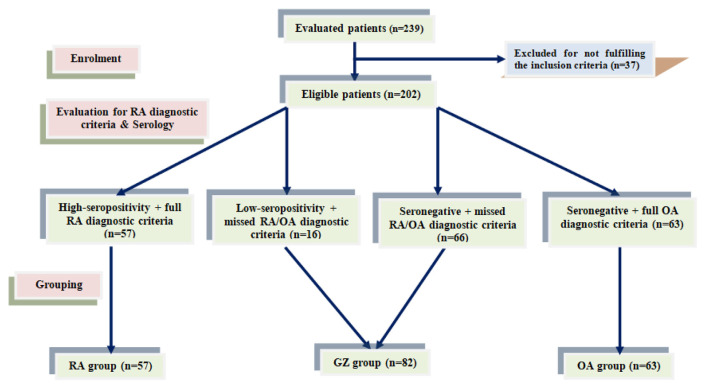
Study flow chart.

According to the predetermined inclusion criteria, 37 patients were excluded and 202 patients were evaluated for the diagnostic criteria of RA and differentiated according to seropositivity for Rheumatic factor (RF) and anti-citrullinated protein antibodies (ACPA) as follows: High RA-seropositive patients (n = 57; 28.2%) and fulfilling the whole diagnostic spectrum of RA (RA-group). Low-seropositive patients who missed some of the diagnostic criteria for both RA and OA were grouped as Low RA-seropositive GZ patients (n = 16; 7.8%). RA-seronegative patients who showed the full diagnostic spectrum for OA were grouped as the OA group (n = 63; 31.2%). RA-seronegative patients who did not fulfill the diagnostic criteria for either RA or OA were grouped as RA-seronegative GZ patients (n = 66; 32.8%), and these 82 patients were grouped as the GZ group (Table 1, Fig. 1).

Patients of the OA group were significantly (P < 0.001) older than patients of other groups, who showed non-significant differences. Patients’ distribution according to gender and their BMI showed insignificant differences between the three groups. Mean duration of disease was significantly shorter for GZ-patients than RA-patients (P < 0.001) and OA-patients (P = 0.003), with insignificantly shorter disease duration of OA-patients than RA-patients (Table 2).

**Fig. 2:**
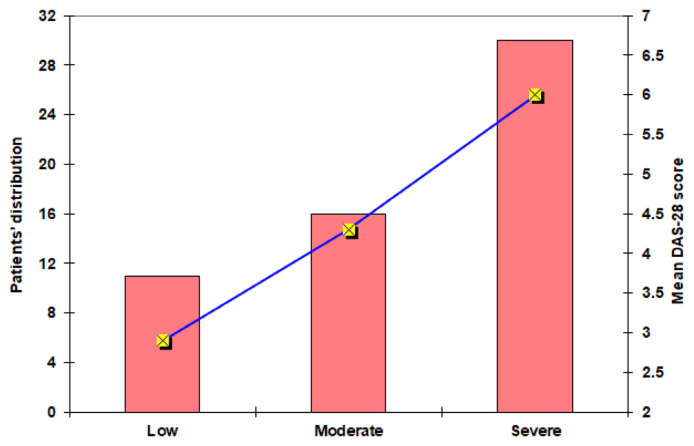
Distribution of RA patients according to DAS-28 score.

According to DAS-28 for RA activity, 30 patients (52.6%) showed high activity with a mean score of 6 (±1.2), 16 patients (28.1%) showed moderate activity of 4.3 (±1.2) mean score and 11 patients (19.3%) showed low activity and 2.9 (±0.9) mean score. The distribution of RA patients according to the DAS-28 and the score for each category are shown in Fig. 2.

**Fig. 3:**
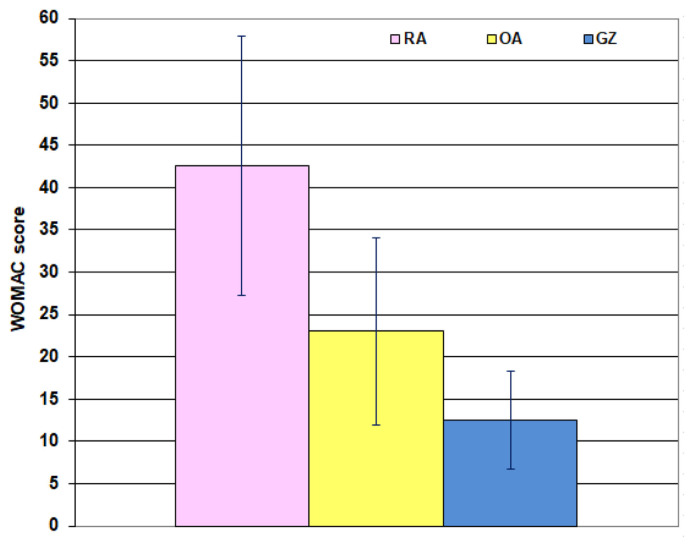
Mean (±SD) value of WOMAC score of the studied arthritis patients.

The determined NRS pain scores were significantly (P < 0.001) lower in GZ patients than scores of patients of other groups who showed insignificant differences (P = 0.219). The frequency of crippled patients and those who had a severe disability was significantly higher among the RA group than other groups, with significantly higher frequency among the OA group than the GZ group. Both RA and OA deleteriously impacted the affected patients as manifested by the highly significant ODI and WOMAC scores compared to scores of GZ patients with significantly higher scores of RA patients than OA patients (Table 3, Fig. 3).

The mean fold change (FC) of PGEL of miR-146a in patients’ samples was significantly higher than in controls’ samples, irrespective of the clinical diagnosis. Samples showed high seropositivity for RF/ACPA (RA-patients) had significantly higher FC of PGEL of miR-146a than samples of OA-patients (P = 0.0002) and of GZ patients (P < 0.001) that showed RF/ACPA seronegativity, while the difference was insignificant (P = 0.381) versus FC of PGEL of miR-146a in samples of GZ patients that showed low RF/ACPA seropositivity. Samples of patients showing RF/ACPA low seronegativity in the GZ group had significantly lower FC of PGEL of miR-146 than those of the OA group (P = 0.028) and samples of GZ patients showed low RF/ACPA seropositivity (P = 0.0004).

The estimated PGEL of miR-21b in patients’ samples was significantly higher than in control samples. The highest expression levels were detected in samples of RA patients that showed significantly higher expression levels in comparison to samples of OA patients (P = 0.00001) and samples of RF/ACPA seronegative GZ patients (P < 0.001), while the difference was insignificant (P = 0.105) in comparison to expression levels in GZ patients who showed low RF/ACPA seropositivity. Expression levels of miR-21b in RF/ACPA seronegative GZ patients were significantly (P = 0.018) lower than levels estimated in samples of low RF/ACPA seropositive GZ patients (Table 4).

**Fig. 4:**
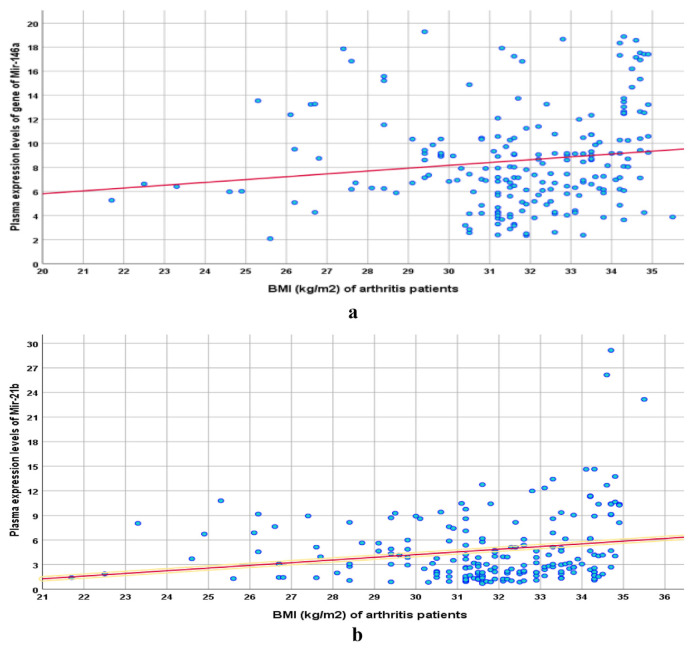
a: Correlation between arthritis patients’ BMI and PGEL of miR-146a. b: Correlation between arthritis patients’ BMI and PGEL of miR-21b.

**Fig. 5:**
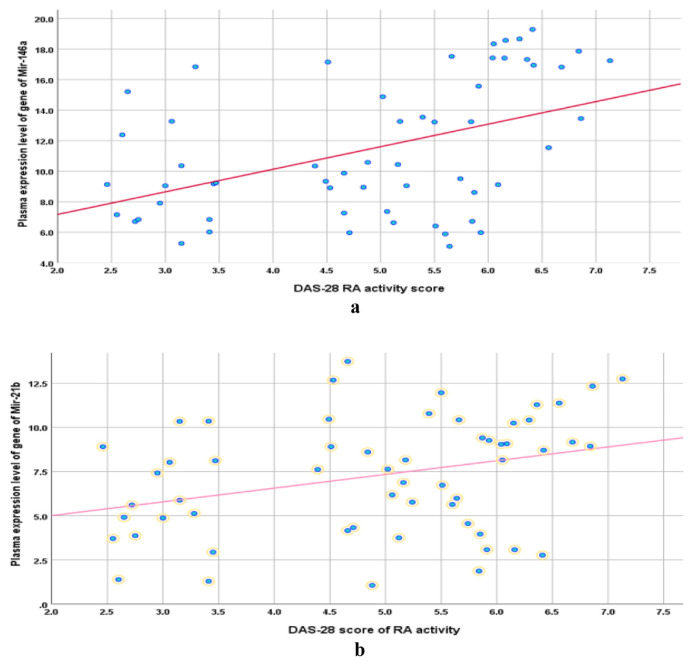
a: Correlation between DAS-28 RA activity score and PGEL of miR-146a. b: Correlation between DAS-28 RA activity score and PGEL of miR-21b.

Among arthritis patients, the estimated FC of PGEL of miR-146a and miR-21b were positively correlated with patients’ BMI (Fig. 4a&b). Among patients showing high seropositivity, DAS-28 RA-activity scores were positively correlated to the PGEL of miR-146a and 21b (Fig. 5a&b). Among arthritis patients who missed some of the RA or OA diagnostic criteria (GZ-patients), low seropositivity was positively correlated with PGEL of miR-146a and 21b (Table 5).

**Fig. 6:**
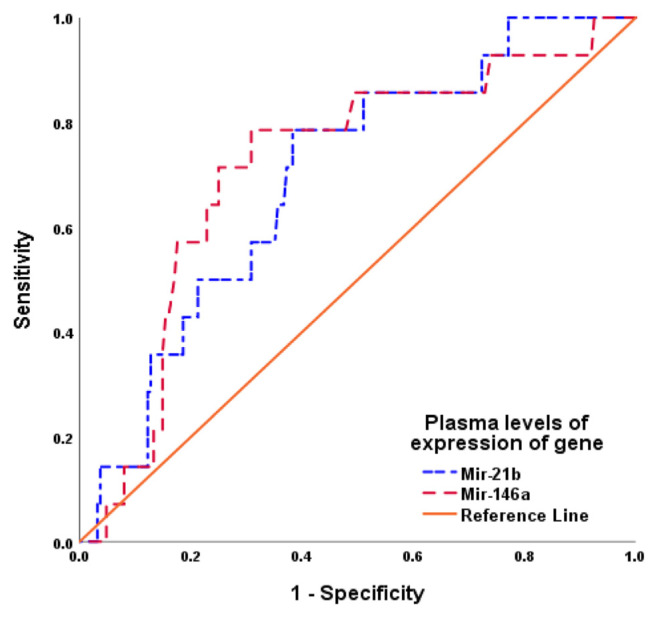
ROC curve analysis of PGEL of miR-146a and miR-21b for prediction of RA among arthritis patients.

Using the ROC curve analysis, the PGEL of miR-146a and miR-21b could predict high-seropositivity among the studied arthritis patients with high AUCs (Fig. 6) and paired-sample analysis for the difference between areas under the ROC curves showed insignificant difference between AUCs of both microRNAs. However, Multivariate regression analysis defined high PGEL of miR-146a as the most significant predictor for high seropositivity and thus might be used to identify RA patients among arthritis patients (Table 6). For the prediction of RA disease activity as judged by the DAS-28 score, regression analysis defined high PGEL of miR-146a as the significant predictor for high disease activity scores with β = 0.456, P < 0.001 (see Table 7).

**Fig. 7:**
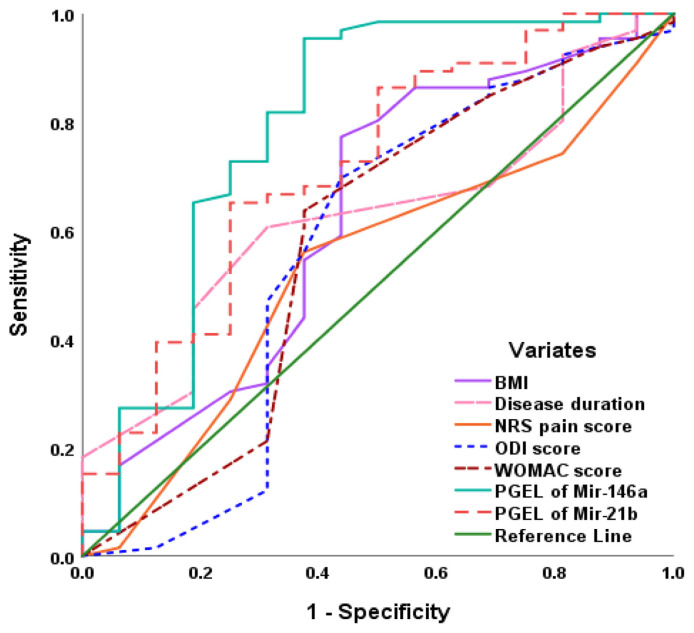
ROC curve analysis of clinical data and PGEL of miR-146a and miR-21b for distinguishing seronegative patients among arthritis patients who did not fulfill the inclusion criteria for a certain type of arthritis.

ROC curve analysis showed that high PGEL of miR-146a could differentiate between RF/ACPA seronegative patients who did not fulfill the diagnostic criteria of RA or OA from patients showing low RF/ACPA seropositivity with the highest AUC among the evaluated variates (Fig. 7). Paired-sample difference analysis showed an insignificantly higher AUC for miR-146a than miR-21b (AUC difference = 0.068 ± 0.386, 95%CI: [−0.260–0.124, P = 0.486) and regression analysis assured the high distinguishing ability of expression levels of miR-146a for cases free of RA (β = 0.382, P < 0.001).

## 18. Discussion

Evaluations of arthritic patients using the well-known scoring systems showed shortcomings for differential diagnosis of cases that were not fulfilling the full diagnostic spectrum for RA or OA; the diagnostic gray-zone (GZ) because of the dependence on findings that are common for various inflammatory conditions as determination of ESR or estimation of serum CRP and the reliance on the disease duration, thus it was mandatory to find another parameter to differentiate these patients according to the type of arthritis. In support of this suggestion, a recent meta-analysis concluded that early-stage KOA is variably defined in the published literature and was mostly evaluated using Kellgren–Lawrence grades of ≥2, which defines the established or later-stage OA leading to under-diagnosis of such cases and indicated the necessity to develop and validate special classification criteria or diagnostic procedures for early-stage KOA [22].

The present study detected significantly elevated PGELs of miR-146a and miR21b in blood samples of arthritis patients than in samples of control non-arthritic subjects. These findings indicated the presence of a relation between deregulated expression levels of micro-RNA and the development or progression of arthritis, and go in hand with previous studies that detected similar relations between arthritis and deregulated levels of multiple micro-RNAs [23–26]. Moreover, the obtained results supported the previous studies that detected upregulation of circulating miR-21b and 146a levels in RA patients than in healthy control and considered estimation of the levels of these microRNAs as the most promising non-invasive biomarkers for the detection of RA [27–32].

The estimated PGELs of miR-146a and 21b were significantly higher in samples of high-seropositive patients (RA patients) than in samples of seronegative patients who fulfilled the OA diagnostic criteria (OA patients). Similarly, **Ciechomska et al**. [29] detected significantly elevated miR-146a expression in serum and synovial fluid of RA patients than in OA patients and **Erfan et al**. [30] detected significantly different miR-146a fold change between the RA and OA groups and concluded that miR-146a could is a promising biomarker for the differentiation between RA and OA.

Among RA patients, the estimated PGELs of miR-146a and 21b were positively correlated with the DAS-28 score, thus changes in levels of these microRNAs might be used to assess RA activity and the response to treatment. These findings are in line with the previously detected significant correlation between miR-146a expression in serum of RA-patients with clinical parameters including DAS28 and with CRP and ESR levels [29,33], with RF, ACCP and Simple Disease Activity Index scores [31,34] and with visual analogue scale and Modified Health Assessment Questionnaire [32]. Also, **Yang et al**. [21] found that miR-21 levels correlate with RA risk and disease activity and its decreased levels are associated with better treatment outcomes and suggested its use as a potential biomarker to monitor RA disease activity and treatment outcome. Also, **Mucientes et al**. [35] found that miR-146a can predict the response to methotrexate and anti-TNF therapies.

Estimated PGELs of miR-146a and −21b were positively correlated with BMI of arthritis patients, similarly, **Andonian et al**. [36] reported an association between the expression of miR-21 and -146a with both visceral adiposity and with thigh intra-muscular adiposity for miR-146a and suggested that microRNAs associated with RA disease activity are more reflective of RA adiposity and impaired metabolism. Thereafter, **Auer et al**., [37] found obesity and RA share dysregulated expression of miR-21 and 146a and attributed this relation to epigenetic alterations caused by obesity that were mediated by non-coding RNAs and increases the susceptibility to autoimmune and inflammatory pathways.

The ROC curve analysis showed significant predictability of PGELs of miR21b and 146a for high seropositivity, the diagnostic hallmark for RA; however, Regression analysis defined high PGELs of miR-146a as the highly significant predictor for the presence of RA and could define RA patients with high disease activity more significantly than miR-21. In line with these findings, **Chen et al**. [33] using ROC curve analysis assured the diagnostic potential of miR-146a for RA-diagnosis with 87% and 86.2% sensitivity and specificity rates. Also, **Safari et al**., [38] detected 96% sensitivity and 86% specificity rates for elevated PGELs of miR-146a for RA diagnosis and concluded that increased expressions of miR-146a in RA patients indicated its involvement in disease incidence and progression, and can play an essential role in the diagnosis and treatment of RA-disease. Recently, **Mahi et al**., [34] using ROC curve analysis showed 85% sensitivity and 100% specificity for miR-146a for RA diagnosis.

Among patients of the Gray-zone, the levels of both microRNAs were significantly higher in low-seropositive than seronegative patients, and so could differentiate between early RA patients and OA patients. However, statistical analyses defined miR-146a as the significant differentiating parameter for patients with OA and early RA patients.

In line with these findings; **Filková et al**. [39] documented that differential expression of circulating miR-146a may characterize the early stage of RA disease. Also, **Anaparti et al. (2017)** [40] concluded that systematically altered circulating miR-146a expression levels in first-degree relatives could identify those at risk before RA onset. Recently, **Chang et al** [41] documented that combined estimation of miR146 expression levels and detection of ACCP could allow accurate diagnosis and prognosis, especially for seronegative patients. Thereafter, **Mahi et al**., [34] concluded that miR-146a can be used as a useful biomarker for early detection of RA diagnosis among other types of arthritis.

The proinflammatory RA progression was attributed to the significantly upregulated expression of miR-146a in the IL-17 producing T cells and in RA than OA synovium [42], to tumor necrosis factor-α (TNF-α)-induced overexpression of miR146a in T cells and suppression of Jurkat T cell apoptosis through gene expression of Fas-associated factor 1, which is a miR-146a-regulated gene and is involved in modulating T cell apoptosis [43], to the reduction of the anti-inflammatory retinoic acid receptor-α transcript by overexpression of miR-146a [29], to high expression levels of miR-21b, a miR that was associated with the inflammatory process, in osteoclast precursors [44], in differentiated adipocytes, which also secreted it [45] and to TNFα induced miR-146a overexpression in human adipocytes [46].

## 19. Conclusion

Early arthritis that does not fulfill the diagnostic spectrum of a certain type of arthritis is not uncommon, leads to missed diagnoses and challenges therapeutic decision-making. Estimation of PGEL of MicroRNA-146a might help the physician to reach the diagnosis and differentiation between early RA and early OA. Moreover, for RA patients, a high PGEL of miR-146a helps to stratify patients according to disease activity and severity.

## 20. Recommendations

The estimation of plasma levels of other micro-RNAs and inflammatory cytokines is recommended to evaluate the interplay between these parameters for initiation of early arthritis.

## Supplementary Information



## Figures and Tables

**Table 1:** Patients’ distribution according to the presence of RA diagnostic criteria.

Scoring of		RA-group (n = 57)	OA-group (n = 63)	GZ-group (n = 82)
**Number of tender joints**	0	0	41 (65.1%)	49 (59.8%)
	1	16 (28.1%)	22 (34.9%)	33 (40.2%)
	2	31 (54.4%)	0	0
	3	8 (14%)	0	0
	5	2 (3.5%)	0	0
**Number of swollen joints**	0	8 (14%)	46 (73%)	42 (51.2%)
	1	30 (52.7%)	17 (27%)	40 (48.8%)
	2	15 (26.3%)	0	0
	3	4 (7%)	0	0
	5	0	0	0
**Serology**	0	4 (7%)	63 (100%)	66 (80.5%)
	2	39 (68.4%)	0	16 (19.5%)
	3	14 (24.6%)	0	0
**Acute-phase reactants**	0	0	37 (58.7%)	36 (43.9%)
	1	57 (100%)	26 (41.3%)	46 (56.1%)
**Duration of disease**	0	0	0	0
	1	57 (100%)	63 (100%)	82 (100%)
**Total score**	Mean	7.3 (±1.6)	2 (±0.8)	2.8 (±0.9)
	Range	6–13	1–4	1–5

**Table 2:** Patient enrolment data.

		RA (n = 57)	OA (n = 63)	GZ (n = 82)	P1	P2	P3
**Age (years)**		47.7 ± 4.9	54.5 ± 5.6	48 ± 7.7	<0.001	0.796	<0.001
**Gender**	Males	23 (40.4%)	25 (39.7%)	31 (37.8%)	0.941	0.762	0.818
	Females	34 (60.6%)	38 (60.3%)	51 (62.2%)			
**BMI (kg/m** **^2^** **)**		31.4 ± 2.7	32.2 ± 2	32.1 ± 1.6	0.064	0.058	0.913
**Duration of disease (years)**	<5	8 (14%)	11 (17.5%)	23 (28%)	0.376	<0.001	0.034
	5–9	26 (45.6%)	36 (57.1%)	51 (62.2%)			
	10–14	18 (31.6%)	13 (20.6%)	8 (9.8%)			
	≥15	5 (8.8%)	3 (4.8%)	0			
	Mean (±SD)	8.8 ± 3.8	7.7 ± 3.3	6.2 ± 2.5	0.057	<0.001	0.003

**Table 3:** Pain & Disability scorings of patients of the three groups.

		RA (n = 57)	OA (n = 63)	GZ (n = 82)	P1	P2	P3
**NRS pain score**		4.5 ± 1.07	4.8 ± 1.65	3.5 ± 1.33	0.219	<0.001	<0.001
**ODI score**	Minimal (0–20)	1 (1.8%)	21 (33.3%)	67 (81.7%)	<0.001	<0.001	<0.001
	Moderate (20–40)	25 (43.8%)	36 (57.1%)	15 (18.3%)			
	Sever (40–60)	18 (31.6%)	5 (8%)	0			
	Crippled (60–80)	13 (22.8%)	1 (1.6%)	0			
	Mean (±SD)	46.1 ± 15.4	27.7 ± 11.8	16.9 ± 6.6	<0.001	<0.001	<0.001
**WOMAC score**		42.55 ± 15.3	23 ± 11	12.56 ± 5.8	<0.001	<0.001	<0.001

**Table 4:** Mean levels of fold-change in the PGEL of miR-146a & 21b in samples of the studied patients who were categorized according to the RF/ACPA serological diagnosis.

		Levels	P1	P2	P3	P4
**Plasma gene-expression level of miR-146a**						
**Healthy control (n = 20)**		1.418 (±0.61)				
**RA-patients (high seropositive; n = 57)**		7.26 (±3.27)	<0.001			
**OA-patients (seronegative; n = 63)**		4.27 (±4.98)	0.012	0.0002		
**GZ patients**	Total (n = 82)	3.45 (±3.63)	0.015	<0.001	0.253	
	Seronegative (n = 66)	2.77 (±2.31)	0.012	<0.001	0.028	
	Low seropositive (n = 16)	6.25 (±6.14)	0.0013	0.381	0.181	0.0004
**Plasma gene-expression level of miR-21b**						
**Healthy control (n = 20)**	1.094 (±0.52)					
**RA-patients (high seropositive; n = 57)**	25.44 (±22.27)	<0.001				
**OA-patients (seronegative; n = 63)**	11 (±11)	0.0001	0.00001			
**GZ patients**	Total (n = 82)	10.5 (±10)	0.0001	<0.001	0.782	
	Seronegative (n = 66)	9.25 (±8.27)	0.00003	<0.001	0.306	
	Low seropositive (n = 16)	15.76 (±14.31)	0.00006	0.105	0.151	0.018

**Table 5:** Correlation between PGEL of miR-146a and miR-21b and patients’ data.

			“r”	P
**Among arthritis patients**	BMI vs.	miR146a	0.147	0.037
		miR21b	0.193	0.006
**Among RA patients**	DAS-28 score	miR146a	0.456	<0.001
		miR21b	0.322	0.015
**Among GZ arthritis patients**	RF/ACPASeronegativity vs.	BMI	−0.262	0.017
		miR146a	0.305	0.005
		miR21b	0.210	0.058

**Table 6:** Statistical analyses for PGEL of miR-146a and miR-21b to define high seropositive patients among arthritis patients.

Receiver Operating Characteristic (ROC) curve					Regression analysis			
	
	AUC	Std. Error	P	95% CI	Type	Variate	β	P
**miR146a**	0.716	0.070	0.007	0.579–0.852	**Univariate**	miR146a	0.328	<0.001
**miR-21b**	0.696	0.064	0.015	0.570–0.822		miR-21b	0.206	0.002
**AUC difference**	0.020	0.374	0.848	[−0.226]−0.186	**Multivariate**	miR-146a	0.372	<0.001

**Table 7:** Statistical analyses for clinical scorings and PGEL of miR-146a and 21b to define seronegative patients among arthritis patients.

Receiver Operating Characteristic (ROC) curve					Regression analysis		
	
	AUC	Std. Error	P	95% CI		β	P
**BMI**	0.628	0.085	0.114	0.460–0.795	**BMI**	0.078	0.494
**Duration of disease**	0.627	0.071	0.117	0.488–0.766	**Duration of disease**	0.148	0.155
**NRS score**	0.530	0.080	0.712	0.374–0.686	**NRS score**	0.005	0.964
**ODI score**	0.566	0.096	0.413	0.377–0.755	**ODI score**	0.093	0.369
**WOMAC score**	0.578	0.091	0.334	0.399–0.757	**WOMAC score**	0.098	0.346
**miR-146a**	0.716	0.075	0.008	0.570–0.862	**miR-146a**	0.382	<0.001
**miR-21b**	0.648	0.073	0.068	0.504–0.792	**miR-21b**	0.199	0.057
**AUC difference**	0.068	0.386	0.486	[−0.260]−0.124			

## Data Availability

Data is available when requited.
