# Supplementary material for: Plasma levels of miR-21b and miR-146a can discriminate rheumatoid arthritis diagnosis and severity
Source: Biomedicine (Taipei). 2025 Mar 1;15(1):30–41. doi: 10.37796/2211-8039.1637 (PMC11959981; doi:10.37796/2211-8039.1637)
Supplement: Supplementary file 1 [file bmed-15-01-030-s001.docx]

**Appendix (1)**

1. Diagnostic criteria for OA according to the U.K. NICE 2014 Guidelines for Osteoarthritis of the Knee, which entails the following
   - Patient was ≥45 years
   - Patient has movement-related joint pain
   - Patient has no
     - Morning knee stiffness lasting ≥30 min to ease  Acute knee injury in the last 6-m
     - Hot and swollen knee or rapidly deteriorating ^(15)^.
2. Patients had at least 1 joint with clinical synovitis with tender and/or swollen joint that was not explained by another disease were considered as RA. Diagnosis was confirmed if the total score according to the 2010 RA classification criteria was ≥6/10 ^(13)^

| Items | Score |
| --- | --- |
| Joint involvement (Tender/Swollen) One large joint | 0 |
| 2-10 large joints | 1 |
| 1-3 small joints ± large joints involvement | 2 |
| 4-10 small joints ± large joints involvement | 3 |
| > 10 joints including at least 1 small joint | 5 |
| Serology  Negative Rheumatic factor (RF) & Anti-citrullinated protein antibodies (ACPA) | 0 |
| Low-positive RF/low-positive ACPA | 2 |
| High-positive RF/high-positive ACPA | 3 |
| Acute-phase reactants  Normal C-reactive protein (CRP) & Erythrocyte sedimentation rate (ESR) | 0 |
| Abnormal CRP & ESR | 1 |
| Duration of symptoms  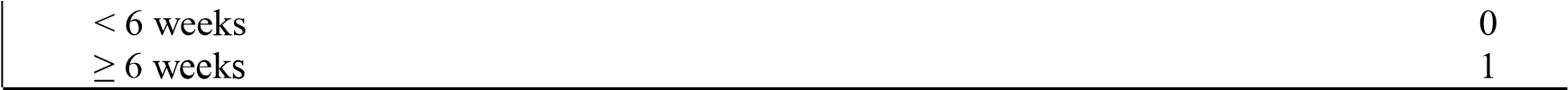 |  |

1. Disease activity score-28 (DAS28), which evaluates the number of tender and swollen joints, estimated ESR or CRP levels and evaluation of the global assessment of health on a visual analog scale of 0 to 10 points with higher points indicate bad global health **^(14)^**. The calculated score was interpreted as follows

| DAS-28 score | Significance |
| --- | --- |
| <2.6 | Remission |
| 2.6 to <3.2 | Low activity |
| 3.2 to <5.1 | Moderate activity |
| ≥5.1 | High activity |

1. The numeric rating scale (NRS) is an 11-point scale with 0 indicating no pain and 10 indicating the worst pain imaginable and was used to evaluate the pain severity of RA or OA ^(16)^.
2. Oswestry Disability Index (ODI) is a 10-item questionnaire that was used to assess the disability secondary to pain or limited mobility caused by RA or OA. Each item was scored by 0-5 according to increased disability and the total ODI score was calculated. Disability was graded as minimal (ODI= 0–20), moderate (ODI=20–40), severe (ODI= 40–60), crippled (ODI score = 60-80) and ODI score of 80–100 indicating that the patient is either bed-bound or exaggerating his or her symptoms ^(17)^.
3. The Western Ontario and McMaster Universities Arthritis Index (WOMAC) was used to evaluate the impact of arthritis on patients' quality of life. WOMAC is a self-administered 3-domain; pain, stiffness, and physical function questionnaire including 24-item, and each were scored by 0-4 points for a maximum score of 96 was presented as 100% and the higher the WOMAC score the worst the impact of arthritis on patients' quality of life ^(18)^.
